# A Cross-Sectional Study on the Prevalence and Risk Stratification of Chronic Kidney Disease in Cardiological Patients in São Paulo, Brazil

**DOI:** 10.3390/diagnostics13061146

**Published:** 2023-03-16

**Authors:** Farid Samaan, Bruna Bronhara Damiani, Gianna Mastroianni Kirsztajn, Ricardo Sesso

**Affiliations:** 1 Research Division, Dante Pazzanese Cardiology Institute, São Paulo 04012-909, SP, Brazil; bruna.damiani@dantepazzanese.org.br; 2 Nephrology Division, Universidade Federal de São Paulo, São Paulo 04023-900, SP, Brazil; gm.kirsztajn@unifesp.br (G.M.K.); rsesso@unifesp.br (R.S.)

**Keywords:** epidemiology, chronic kidney disease, cardiovascular diseases, cardiac care facilities

## Abstract

Chronic kidney disease (CKD) provides a worse prognosis for patients with heart disease. In Latin America, studies that analyzed the prevalence and risk stratification of CKD in this population are scarce. We aimed to evaluate CKD prevalence and risk categories in patients of a public referral cardiology hospital in São Paulo, Brazil. This was a cross-sectional study based on a laboratory database. Outpatient serum creatinine and proteinuria results performed between 1 January 2021 and 31 December 2021 were analyzed. CKD was defined by estimated glomerular filtration rate (eGFR) <60 mL/min/1.73 m^2^ and proteinuria, by the albumin/creatinine ratio in a spot urine sample (UACR) >30 mg/g. A total of 36,651 adults were identified with serum creatinine levels (median age 72.4 [IQR, 51.0–73.6] years, 51% male). Among them, 51.9% had UACR dosage (71.5% with UACR < 30 mg/g, 22.6%, between 30–300 mg/g, and 5.9% with UACR > 300 mg/g). The prevalence of CKD was 30.9% (15.3% stage 3a, 10.2% stage 3b, 3.6% stage 4, and 1.7% stage 5), and the distribution of patients in the risk categories of the disease was: 52.0% with low-risk, 23.5%, moderate risk, 13.0%, high risk, and 11.2%, very high. In an outpatient setting, the prevalence of CKD in cardiological patients was almost three times (31%) that of the general population; about half of the individuals evaluated (48%) were not screened for an important risk marker (proteinuria), and approximately a quarter of these patients (24%) were in the high or very high CKD risk categories.

## 1. Introduction

Chronic kidney disease (CKD) is a public worldwide health problem affecting 10–14% of the adult population, including 12% of people with hypertension, 15% with diabetes, and 30% of the elderly [1]. The World Health Organization defined this condition as the world’s most neglected non-communicable disease [2]. As an oligosymptomatic disease in its early stages, CKD is globally underdiagnosed [3]. Moreover, most countries do not have national systems for surveillance and assistance in all stages of CKD [2,3]. 

Brazilian and international guidelines recommend conducting serum creatinine and urinary protein testing, at least once a year, for patients at risk of CKD [4,5]. These two tests are simple, inexpensive, and widely available in the Brazilian Unified Health System and in most countries. Nevertheless, studies have shown that less than 6% of patients with CKD are diagnosed in the early stages of the disease [3]. In addition, only 25% of CKD-risk patients undergo adequate screening in primary health care, and 40% of cases are lately referred to a nephrologist [6,7].

Among potentially dangerous comorbidities for CKD, heart diseases stand out due to their high prevalence in the general population [8]. CKD has a high impact on the prognosis of patients with coronary artery disease, heart valve diseases, and chronic cardiomyopathies [9,10,11]. In addition, proteinuria is an independent risk factor for hospitalization due to heart failure and death, even in patients with preserved glomerular filtration rate [12].

Thus, considering that the detection of silent CKD through laboratory records is feasible and can produce important information to scale prevention programs [13,14,15], this study aimed to evaluate the CKD prevalence and risk categories in patients of a public referral cardiology hospital.

## 2. Materials and Methods

### 2.1. Study Design and Reference Population

This retrospective cross-sectional study is based on records of laboratory tests performed between 1 January 2021 and 31 December 2021 in a public hospital in São Paulo city, Brazil. This hospital is one of three tertiary cardiology referral services for a metropolitan region with 20 million inhabitants, performing alone annually about 200,000 outpatient consultations, 25,000 emergency consultations, 8000 hemodynamic procedures, 2000 cardiac surgeries, and 5000 intra-hospital hemodialysis sessions [16].

Only laboratory results performed on adults at the outpatient level were analyzed, excluding test results from patients under 18 years old and those performed on hospitalized patients. This study was performed following the Declaration of Helsinki and was approved by the institutional research ethics committee under the certificate of presentation of ethical assessment number 58300122.9.0000.5462.

### 2.2. Data Source, Variables, and Definitions

Participants’ demographic data, such as gender and date of birth, and serum creatinine and urinary protein results, were obtained from the laboratory records. From the serum creatinine level, age, and gender, the estimated glomerular filtration rate (eGFR) was calculated using the CKD-EPI equation [17]. The diagnosis of CKD was defined by an eGFR < 60 mL/min/1.73 m^2^ and classified in stages 3a (45–59 mL/min/1.73 m^2^), 3b (30–44 mL/min/1.73 m^2^), 4 (15–29 mL/min/1.73 m^2^), or 5 (<15 mL/min/1.73 m^2^) following international guidelines [5]. The lowest value of creatinine level was used for CKD classification in patients with two or more dosages of creatinine in one year to avoid overestimating the prevalence of CKD. The ethnicity variable was not assessed in the CKD-EPI equation due to the unavailability of race data, the high degree of miscegenation in the Brazilian population, and previous study reporting that the adjustment of race data does not add accuracy for estimating renal function in this population [18].

The proteinuria was evaluated by the albumin/creatinine ratio in a spot urine sample (UACR) since this is a routine test contained in the institutional protocol for the admission of adults and having the best sensitivity and specificity for the screening of CKD [5]. Proteinuria was defined by UACR > 30 mg/g and categorized into the levels mild or absent (<30 mg/g), moderate (30–300 mg/g), or severe (>300 mg/g) following current guidelines [4,5].

According to the CKD risk map, which considers eGFR ranges and the albuminuria categories, participants were classified as having low, moderate, high, or very high CKD risk [5]. Patients at low CKD risk show eGFR > 60 mL/min/1.73 m^2^ and UACR < 30 mg/g. Moderate risk is determined by conditions with eGFR > 60 mL/min/1.73 m^2^ and UACR between 30 and 300 mg/g, or eGFR between 45 and 59 mL/min/1.73 m^2^ and UACR < 30 mg/g. The high CKD risk category includes patients having any of the following combinations: eGFR > 60 mL/min/1.73 m^2^ and UACR > 300 mg/g, eGFR 45–59 mL/min/1.73 m^2^ and UACR 30–300 mg/g, or eGFR 30–44 mL/min/1.73 m^2^ and UACR < 30 mg/g. Values of eGFR 45–59 mL/min/1.73 m^2^ and UACR > 300 mg/g, eGFR 30–44 mL/min/1.73 m^2^ and UACR > 30 mg/g, or eGFR < 30 mL/min/1.73 m^2^ regardless of UACR determine patients at a very high CKD risk.

Finally, patients were grouped according to the main medical subspecialty of the institution’s outpatient clinic where they were treated. Thus, the group classes were coronary heart disease, valvular heart disease, arrhythmias, cardiomyopathies, hypertension, dyslipidemias, and others (vascular surgery, cardiogeriatrics, congenital heart disease, sports medicine, and heart transplantation, among others). The historical series of the most prevalent outpatient consultations were considered for this classification. Hence, patients treated at the coronary heart disease and angioplasty outpatient clinics were grouped into the coronary heart disease category. The patients treated at the electrophysiology and pacemaker outpatient clinics were grouped into the arrhythmias category. Conventionally, patients treated by diverse medical subspecialties in two or more outpatient clinics were categorized by the most frequent specialty of treatment or the first medical visit recorded during the analyzed period. The dates and frequencies of the patients’ medical appointments were obtained from the hospital’s administrative database.

### 2.3. Statistical Analysis

Categorical variables were expressed in frequency and, according to the Kolmogorov–Smirnov normality test result, continuous variables were expressed as mean (standard deviation) or median (interquartile range). Intergroup frequency comparisons were performed using the Chi-square test, while Student’s *t*-test and Mann–Whitney U-test were performed on quantitative variables with normal and non-normal distribution, respectively. Test results were considered statistically significant when the *p*-value was <0.05. A logistic regression model was applied to evaluate the effect of gender, age, and primary outpatient clinic on variables used in a binary way: eGFR > or <60 mL/min/1.73 m^2^; UACR < or >30 mg/g; and CKD risk categories, high and very high or the others. The evaluated effects were represented by the odds ratio, with 95% confidence intervals. The independent variables were evaluated separately in each univariate model and then together in a multiple regression model. All the statistical analyses were conducted using SPSS software version 18.0 (SPSS Inc., Chicago, IL, USA).

## 3. Results

**Figure 1:**
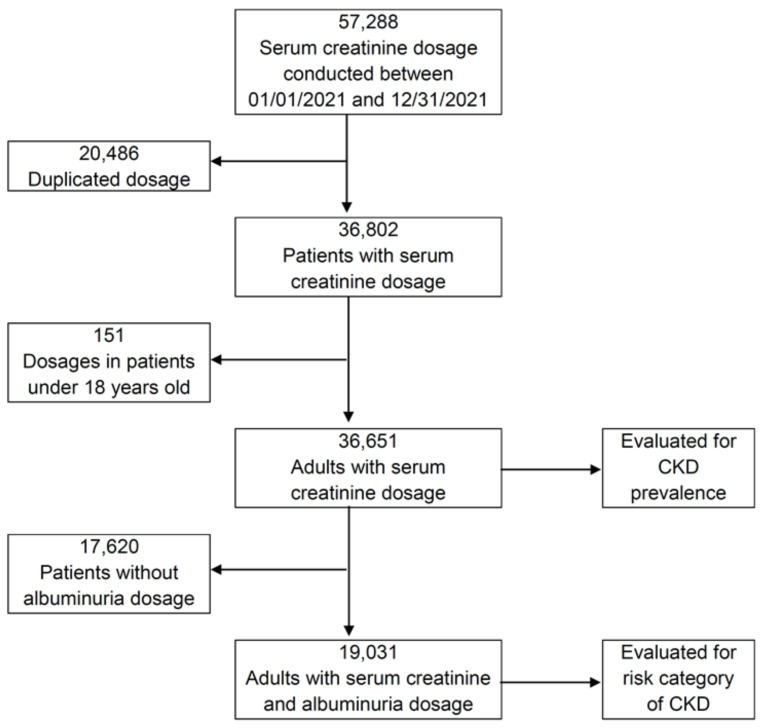
Flowchart depicting the inclusion criteria of participants. CKD, chronic kidney disease.

A total of 57,288 ambulatory dosages of serum creatinine were performed in the studied period. After excluding repeated dosages in the same patient (*n* = 20,486) and dosages in patients under 18 years old (*n* = 151), 36,651 adults were evaluated for CKD prevalence. Among them, 19,031 also had UACR dosages and therefore were evaluated for risk category of CKD (Figure 1).

The patients’ median age was 72.4 years (interquartile range, 51.0–73.6), and 51.3% were males. The most frequent outpatient clinic source of the patients was coronary artery disease (31.6%), followed by valvular heart disease (14.2%), arrhythmias (10.1%), cardiomyopathies (9.4%), hypertension (9.1%) and dyslipidemia (5.8%). Patients classified as belonging to ‘other’ outpatient clinics classification were 19.6% of the total. CKD was detected in 30.9% of the participants, of which 15.3% were in stage 3a, 10.2% in stage 3b, 3.6% in stage 4, and 1.7% in stage 5. Dosages of UACR were conducted in 51.9% of patients, of which 71.5% had mild or absent albuminuria, 22.6% moderate, and 5.9% had severe proteinuria. Thus, according to the CKD risk categories, in 52.0% of the patients, the risk was low, 23.5% moderate, 13.0% high, and 11.2% very high (Table 1).

The UACR dosage was significantly more frequent in females (53.2%) than in males (50.7%) (*p* < 0.001). Regarding the participants’ age group, the frequency of UACR dosage was higher the older their age group (55.5% in >75 years, 53.0% in 60–74 years, 50.5% in 45–59 years, 36.8% in 30–44 years, and 24.7% in 18–29 years ‘group) (*p* < 0.001). The percentage of UACR requests was higher in the hypertension outpatient clinic (81.3%), followed by dyslipidemia (76.4%), cardiomyopathies (61.9%), coronary artery disease (49.4%), arrhythmias (46.8%) and valvular heart disease (38.4%) groups (*p* < 0.001) (Table 2).

Reduced eGFR (eGFR < 60 mL/min/1.73 m^2^) was more frequent in females (31.7%) than in males (30.1%) and in the elderly age groups (56.8% in >75 years, 30.7% in 60–74 years, 25.5% in 45–59 years, 8.4% in 30–44 years, and 2.5% in 18–29 years’ group). Cardiomyopathies was the outpatient clinic category with the highest percentage of patients showing reduced eGFR (32.4%), followed by arrhythmias (31.4%), hypertension (31.2%), coronary artery disease (29.6%), dyslipidemia (29.2%), and valvular heart disease (27.3%) clinics (Table 3 and Table S1).

Albuminuria (UACR > 30 mg/g) was more frequent in men (30.1%) than in women (26.9%) and in the elderly age groups (36.9% in >75 years, 28.5% in 60–74 years, 26.0% in 45–59 years, 22.4% in 30–44 years, and 23.3% in 18–29 years’ group). The outpatient clinic category with the highest percentage of patients with albuminuria was dyslipidemia (32.7%), followed by valvular heart disease (31.9%), coronary heart disease (28.7%), hypertension (28.0%), arrhythmias (24.1), and cardiomyopathies (22.8%) (Table 3 and Table S2).

**Figure 2:**
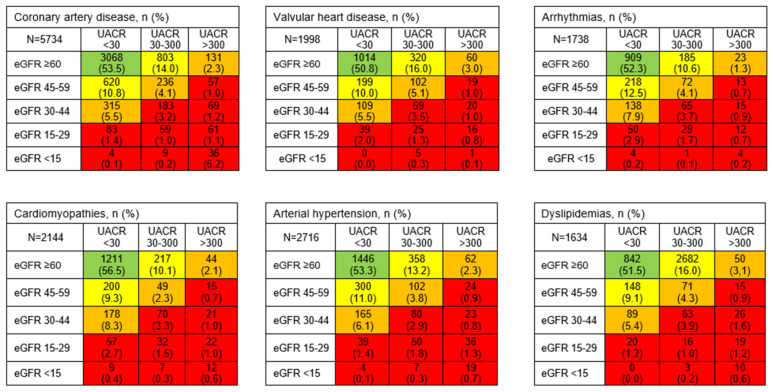
Distribution of patients concerning the chronic kidney disease risk map [5] and main outpatient clinic (*n* = 19,031). UACR, albumin/creatinine ratio in spot urine sample (mg/g); eGFR, estimated glomerular filtration rate (mL/min/1.73 m^2^). Colors indicate the risk categories of chronic kidney disease: green, low risk; yellow, moderate risk; orange, high risk; red, very high risk.

Similar percentages of high or very high risk of CKD were observed between men (24.1%) and women (24.2%). High or very high-risk categories were more frequent in the elderly age groups (41.9% in >75 years, 23.7% in 60–74 years, 20.2% in 45–59 years, 7.8% in 30–44 years, and 3.4% in 18–29 years’ group). The outpatient clinic with the highest percentage of patients showing high or very high CKD risk was arrhythmias (24.5%), followed by cardiomyopathies (24.1%), dyslipidemia (23.4%), valvular heart disease (23.3%), hypertension (22.5%), and coronary heart disease (21.7%) (Table 3 and Table S3). The distribution of patients on the CKD risk map is graphically shown in Figure 2.

Each cell contains the absolute number of participants and the corresponding percentage in parentheses. eGFR, estimated glomerular filtration rate; UACR, urinary albumin-creatinine ratio; CKD, chronic kidney disease; CAD, coronary artery disease; VHD, valvular heart disease.

In multiple regression analysis, by testing the variables gender, age group, and outpatient clinic category, the factors independently related to reduced eGFR (<60 mL/min/1.73 m^2^) were female gender, the most advanced age groups, and the outpatient clinics for coronary artery disease, hypertension, arrhythmias, and cardiomyopathies, compared with valvular heart disease (Table S1). Factors associated with UACR > 30 mg/g were male gender, age > 75 years old, and outpatient clinics for hypertension, coronary artery disease, valvular heart disease, and dyslipidemia categories, compared with the cardiomyopathies category (Table S2). The factors associated with the highest risk categories of CKD (high or very high) were the most advanced age groups and outpatient clinics for cardiomyopathies, and arrhythmias categories, compared with the coronary heart disease category (Table S3).

## 4. Discussion

The present study showed that adults of a referral cardiology hospital had a 31% prevalence of CKD based on their eGFR and a 28% prevalence of proteinuria. In 24% of patients tested for proteinuria, the CKD risk category was high or very high. Testing for proteinuria, an important prognostic factor, was not performed in about half of the patients.

Patients with CKD have a higher prevalence of cardiovascular diseases than the general population due to older age and a higher prevalence of hypertension and diabetes mellitus. In addition, non-traditional risk factors for cardiovascular diseases may be present, such as bone mineral disease/vascular calcification, inflammation, oxidative stress, hyperuricemia, and hypervolemia [8,9]. Nevertheless, few studies have been specifically designed to determine the prevalence of CKD in people with heart diseases and their main subtypes, such as coronary heart disease, cardiomyopathies, valvular heart disease, and arrhythmias.

Even considering that the definition of CKD includes people with proteinuria regardless of reduced eGFR, the evaluation of CKD prevalence performed in this study was based only on eGFR of all participants who had a creatinine dosage [5]. It has been recognized worldwide that there is a failure in the screening of CKD by measuring proteinuria [19,20]. Therefore, patients tested for proteinuria are more likely to be sicker and have a higher prevalence of CKD than those not tested. In fact, in our study, participants who had the UACR dosage were older and predominantly female compared to those who did not. Older age and female gender are known risk factors for CKD due to the lower number and nephron mass [21].

Nevertheless, the prevalence of CKD in this study was even higher than the average prevalence reported in previous studies on patients with heart diseases (31% versus 26% [range, 8–38%]), which could be explained by the higher age (72 vs. 61 [range, 42–63] years) and lower frequency of male patients (51% vs. 54% [range, 37–76%]) in the present study than in the previous reports (Table S4) [3,10,22,23,24,25,26,27,28,29,30,31]. On the other hand, the prevalence of proteinuria in our study was lower than the average values observed in previous reports (28% vs. 34% [range, 8–62%]) [3,22,23,24,27,30]. This result could be explained by the lower frequency of male patients in our study than in previous ones since men are known to have a higher chance of presenting proteinuria than women. Gender differences in the prevalence of proteinuria may be related to prescribing patterns, differences in responses and adherence to therapies, as well as hormonal factors [32]. However, as expected, the main factor associated with the prevalence of proteinuria in previous studies was diabetes mellitus [3,10,22,31]. Unfortunately, this association could not be evaluated in our study.

The proteinuria testing rate in our entire cohort was 52%, higher than in previous studies on patients at risk for CKD, such as those with hypertension or diabetes mellitus (3–40%) [19,20,33,34]. This difference could be a consequence of conducting this research in a specialized teaching hospital, where adherence to institutional protocols and guidelines may be higher than in other health care institutions. Even so, as proteinuria is an important risk factor for worsening cardiovascular outcomes, the absence of this screening in about 50% of the participants should be viewed with concern [12,35]. It is well established that late diagnosis of CKD is associated with increased morbidity, mortality, and resource utilization [36]. In fact, both reduced glomerular filtration rate and proteinuria are recognized as worse prognostic factors in the guidelines of scientific societies of cardiology [37,38,39].

The UACR request was higher in the hypertension outpatient clinic and less in the valvular heart disease one. Assuming that all patients with cardiovascular disease are at risk for CKD, this difference could reflect that physicians in the former clinic are more aware of the importance of CKD risk stratification than in the latter one.

Our study indicated, indirectly and with some limitations, that the patients with the highest prevalence of proteinuria and reduced eGFR came from the valvular heart disease and the cardiomyopathies outpatient clinics, respectively. Studies designed to determine the prevalence of CKD considering the subtypes of heart diseases are scarce (Table S4). Thus, it was not possible to contrast these results with others; and they need to be confirmed in further studies specifically designed for this purpose.

The limitations of this study must be acknowledged. First, the interpretation of CKD prevalence estimates must be performed cautiously, considering the data source used (laboratory results) and the retrospective study design. Second, we could not evaluate other characteristics of the participants regarding CKD risk and severity. Hence, comorbidities (e.g., diabetes, hypertension, dyslipidemia, and obesity) and laboratory identification of certain conditions (e.g., anemia and disorders of calcium, phosphorus, and parathyroid) could not be addressed. In addition, many patients are usually seen once a year as part of the typical dynamic of a public, tertiary, and referral hospital. Therefore, we could not apply the three-month interval of creatinine dosages to define CKD [5]. Finally, the adopted methodology does not incorporate a precise definition of the participants’ baseline cardiological diagnosis. Thereby, the diagnostic inferences should be considered with caution. Moreover, the main diagnosis could not be confirmed in patients with more than one cardiovascular condition.

## 5. Conclusions

In an outpatient setting, the prevalence of CKD in cardiological patients was almost three times that of the general population. Moreover, about half of the individuals evaluated were not screened for an important risk marker (proteinuria), and approximately a quarter of these patients were in the high or very high CKD risk categories.

## Figures and Tables

**Table 1:** Descriptive data on participants of the study.

Variable	*n* = 36,651
Age (years)	72.4 (51.0–73.6)
18–29, *n* (%)	243 (0.7)
30–44, *n* (%)	910 (2.5)
45–59, *n* (%)	10,735 (29.3)
60–74, *n* (%)	21,367 (58.3)
>75, *n* (%)	3396 (9.3)
Males, *n* (%)	18,806 (51.3)
Main outpatient clinic	
Coronary artery disease, *n* (%)	11,599 (31.6)
Valvular heart disease, *n* (%)	5202 (14.2)
Arrhythmias, *n* (%)	3711 (10.1)
Cardiomyopathies, *n* (%)	3462 (9.4)
Hypertension, *n* (%)	3340 (9.1)
Dyslipidemias, *n* (%)	2140 (5.8)
Other, *n* (%)	7197 (19.6)
Serum creatinine (mg/dL)	0.95 (0.80–1.56)
eGFR (mL/min/1.73 m^2^)	74.3 (54.4–75.6)
>60, *n* (%)	25,337 (69.1)
45–59, *n* (%)	5615 (15.3)
30–44, *n* (%)	3746 (10.2)
15–29, *n* (%)	1332 (3.6)
<15, *n* (%)	621 (1.7)
Albuminuria/creatinine ^1^ (mg/g)	12.6 (6.2–36.6)
<30, *n* (%)	13,598 (71.5)
30–300, *n* (%)	4304 (22.6)
>300, *n* (%)	1129 (5.9)
Risk category of CKD ^1^	
Low, *n* (%)	9905 (52.0)
Moderate, *n* (%)	4529 (23.8)
High, *n* (%)	2474 (13.0)
Very high, *n* (%)	2123 (11.2)

eGFR, estimated glomerular filtration rate. CKD, chronic kidney disease. ^1^ *n* = 19,031.

**Table 2:** Dosage of albuminuria according to gender, age, and main outpatient clinic of participants.

		Dosage of Albuminuria/Creatininuria, *n* (%)		
		Yes	No	*p*-Value
Gender	Male	9543 (50.7)	9263 (49.3)	<0.001
	Female	9488 (53.2)	8357 (46.8)	
Age group (years)	18–29	60 (24.7)	183 (75.3)	<0.001
	30–44	335 (36.8)	575 (63.2)	
	45–59	5422 (50.5)	5313 (49.5)	
	60–74	11,328 (53.0)	10,039 (47.0)	
	≥75	1886 (55.5)	1510 (44.5)	
Main outpatient clinic	Coronary artery disease	5734 (49.4)	5865 (50.6)	<0.001
	Valvular heart disease	1998 (38.4)	3204 (61.6)	
	Arrhythmias	1738 (46.8)	1973 (53.2)	
	Cardiomyopathies	2144 (61.9)	1318 (38.1)	
	Hypertension	2716 (81.3)	624 (18.7)	
	Dyslipidemias	1634 (76.4)	506 (23.6)	
	Others	3067 (42.6)	4130 (57.4)	
Total		19,031 (51.9)	17,620 (48.1)	

**Table 3:** Estimated glomerular filtration rate, albuminuria, and risk category of chronic kidney disease stratified by gender, age, and main outpatient clinic of participants.

Variable	eGFR (mL/min/1.73 m2)					UACR (mg/g)			Risk Category of CKD			
	≥60	45–59	30–44	15–29	<15	<30	30–300	>300	Low	Moderate	High	Very High
Gender												
Male	13,150 (69.9)	2911 (15.5)	1788 (9.5)	611 (3.2)	346 (1.8)	6679 (69.9)	2225 (23.3)	651 (6.8)	4981 (52.2)	2265 (23.7)	1219 (12.8)	1078 (11.3)
Female	12,187 (68.3)	2704 (15.2)	1958 (11.0)	721 (4.0)	275 (1.5)	6934 (73.0)	2084 (21.9)	478 (5.0)	4924 (51.9)	2264 (23.9)	1255 (13.2)	1045 (11.0)
Age group (years)												
18–29	237 (97.5)	1 (0.4)	1 (0.4)	1 (0.4)	3 (1.2)	46 (76.7)	11 (18.3)	3 (5.0)	46 (76.7)	10 (16.7)	2 (3.3)	2 (3.3)
30–44	834 (91.6)	22 (2.4)	12 (1.3)	10 (1.1)	32 (3.5)	260 (77.6)	56 (16.7)	19 (5.7)	248 (74.0)	61 (18.2)	16 (4.8)	10 (3.0)
45–59	7999 (74.5)	1361 (12.7)	866 (8.1)	310 (2.9)	199 (1.9)	4011 (74.0)	1100 (20.3)	311 (5.7)	3155 (58.2)	1173 (21.6)	597 (11.0)	497 (9.2)
60–74	14,801 (69.3)	3315 (15.5)	2.164 (10.1)	744 (3.5)	343 (1.6)	8091 (71.4)	2553 (22.5)	684 (6.0)	5904 (52.1)	2742 (24.2)	1441 (12.7)	1241 (11.0)
≥75	1466 (43.2)	916 (27.0)	703 (20.7)	267 (7.9)	44 (1.3)	1190 (63.1)	584 (31.0)	112 (5.9)	552 (29.3)	543 (28.8)	418 (22.2)	373 (19.8)
Main clinic												
CAD	8170 (70.4)	1913 (16.5)	1083 (9.3)	329 (2.8)	104 (0.9)	4090 (71.3)	1290 (22.5)	354 (6.2)	3068 (53.5)	1423 (24.8)	682 (11.9)	561 (9.8)
VHD	3783 (72.7)	813 (15.6)	442 (8.5)	143 (2.7)	21 (0.4)	1361 (68.1)	521 (26.1)	116 (5.8)	1014 (50.8)	519 (26.0)	271 (13.6)	194 (9.7)
Arrhythmias	2547 (68.6)	605 (16.3)	401 (10.8)	137 (3.7)	21 (0.6)	1319 (75.9)	352 (20.3)	67 (3.9)	909 (52.3)	403 (23.2)	233 (13.4)	193 (11.1)
Cardiomyopathies	2339 (67.6)	437 (12.6)	413 (11.9)	206 (6.0)	67 (1.9)	1655 (77.2)	375 (17.5)	114 (5.3)	1211 (56.5)	417 (19.4)	271 (12.6)	245 (11.4)
Hypertension	2297 (68.8)	540 (16.2)	325 (9.7)	138 (4.1)	40 (1.2)	1955 (72.0)	597 (22.0)	164 (6.0)	1447 (53.3)	658 (24.2)	329 (12.1)	282 (10.4)
Dyslipidemias	1516 (70.8)	311 (14.5)	230 (10.7)	62 (2.9)	21 (1.0)	1099 (67.3)	415 (25.4)	120 (7.3)	842 (51.5)	410 (25.1)	210 (12.9)	172 (10.5)
Others	4685 (65.1)	996 (13.8)	852 (11.8)	317 (4.4)	347 (4.8)	2119 (69.1)	754 (24.6)	194 (6.3)	1414 (46.1)	699 (15.4)	478 (15.6)	476 (15.5)

## Data Availability

Data supporting the reported results can be obtained on request.

## Supplementary Materials

The following supporting information can be downloaded at: https://www.mdpi.com/article/10.3390/diagnostics13061146/s1. Table S1. Univariate and multivariate analyses of factors related to the reduced estimated glomerular filtration rate (*n* = 36,651). Table S2. Univariate and multivariate analysis of factors related to urine albumin/creatinine ratio >30 mg/g (*n* = 19,031). Table S3. Univariate and multivariate analyses of factors related to the highest risk categories of chronic kidney disease (*n* = 19,031). Table S4. Studies evaluating the prevalence of chronic kidney disease in patients with cardiovascular disorders [3,10,17,22,23,24,25,26,27,28,29,30,31,40,41].
