# Supplementary material for: A Cross-Sectional Study on the Prevalence and Risk Stratification of Chronic Kidney Disease in Cardiological Patients in São Paulo, Brazil
Source: Diagnostics (Basel). 2023 Mar 16;13(6):1146. doi: 10.3390/diagnostics13061146 (PMC10047703; doi:10.3390/diagnostics13061146)
Supplement: Supplementary file 1 [file diagnostics-13-01146-s001.zip › diagnostics-2222983-supplementary.pdf]

A cross-sectional study on the prevalence and risk stratification of chronic kidney disease in a referral cardiology hospital in São Paulo, Brazil.

Supplementary material

Table S1. Univariate and multivariate analyses of factors related to reduced glomerular filtration rate (N=36,651)

Variable	eGFR <60 ml/min/1.73m^2 N (%)	Univariate			Multivariate		
		OR	CI 95%	p-value	OR	CI 95%	p-value
Gender							
Male	5,656 (30.1)	—	—		—	—	
Female	5,658 (31.7)	1.08	1.03-1.13	<0.001	1.04	0.99-1.08	0.14
Age							
18–29	6 (2.5)	—	—		—	—	
30-44	76 (8.4)	3.60	1.68-9.36	0.003	3.80	1.78-9.89	0,002
45 - 59	2,736 (25.5)	13.5	6.57 - 34.3	<0.001	15,0	7.29- 38.0	<0.001
60-74	6,566 (30.7)	17.5	8.53 - 44.4	<0.001	19.5	9.48-49.4	<0.001
>75	1,930 (56.8)	52.0	25.2-132	<0.001	57.0	27.7-145	<0.001
Main outpatient clinic							
Valvular heart disease	1,419 (27.3)	—	—		—	—	
Dyslipidemias	624 (29.2)	1.10	0.98-1.23	0.102	1.01	0.91 - 1.14	0.801
Coronary artery disease	3,429 (29.6)	1.12	1.04 - 1.20	0.003	1.04	0.97-1.12	0.289
Hypertension	1,043 (31.2)	1.21	1.10-1.33	<0.001	1.13	1.03-1.25	0.013
Arrhythmias	1,164 (31.4)	1.22	1.11-1.34	<0.001	1.18	1.08-1.30	<0.001
Cardiomyopathies	1,123 (32.4)	1.28	1.17-1.41	<0.001	1.33	1.20-1.46	<0.001
Other	2,512 (34.9)	1.43	1.32-1.55	<0.001	1.36	1.25-1.47	<0.001
Total	11,314 (30.9)						

eGFR, estimated glomerular filtration rate; OR, Odds ratio; CI, confidence interval

Table S2. Univariate and multivariate analysis of factors related to urine albumin/creatinine ratio >30mg/g (N=19,031)

Variable	UACR > 30mg/g	Univariate			Multivariate		
	N (%)	OR	CI 95%	p-value	OR	CI 95%	p-value
Gender							
Male	2,876 (30.1)	—	—		—	—	
Female	2,561 (27.0)	0.86	0.80-0.91	<0.001	0.83	0.78 - 0.89	<0.001
Age							
18–29	14 (23.3)	—	—		—	—	
30-44	75 (22.4)	0.95	0.49-1.82	0.872	0.95	0.51 - 1.89	0.888
45 - 59	1,411 (26.0)	1.16	0.63-2.11	0,637	1.13	0.63-2.14	0,692
60-74	3,237 (28.6)	1.32	0.72-2.39	0.371	1.29	0.72 - 2.44	0.413
>75	696 (36.9)	1.92	1.05-3.52	0.035	1.87	1.05-3.56	0.044
Main outpatient clinic							
Cardiomyopathies	489 (22.8)	—	—		—	—	
Arrhythmias	419 (24.1)	1.08	0.93-1.25	0.341	1.04	0.89-1.21	0.631
Hypertension	761 (28.0)	1.32	1.15-1.50	<0.001	1.30	1.39-1.48	<0.001
Coronary artery disease	1,644 (28.7)	1.36	1.21 - 1.53	<0.001	1.27	1.13-1.43	<0.001
Other	948 (30.9)	1.51	1.33-1.72	<0.001	1.41	1.24-1.61	<0.001
Valvular heart disease	637 (31.9)	1.58	1.38-1.82	<0.001	1.57	1.36-1.80	<0.001
Dyslipidemias	535 (32.7)	1.65	1.43-1.90	<0.001	1.60	1.39-1.85	<0.001
Total	5,433 (28.6)						

UACR, urine albumin/creatinine ratio; OR, Odds ratio; CI, confidence interval.

Table S3. Univariate and multivariate analyses of factors related to the highest risk categories of chronic kidney disease[5] (N=19,031)

Variable	High or very-high risk of CKD N (%)	Univariate			Multivariate		
		OR	CI 95%	p-value	OR	CI 95%	p-value
Gender							
Male	2297 (24.1)	—	—		—	—	
Female	2300 (24.2)	1.01	0.95-1.08	0.783	0.95	0.89-1.02	0.149
Age							
18–29	4 (6.7)	—	—		—	—	
30-44	26 (7.8)	1.18	0.40-3.51	0.768	1.24	0.42 - 3.37	0.704
45 - 59	1094 (20.2)	3.54	1.28-9.78	0.015	3.93	1.42-10.89	0.008
60-74	2682 (23.7)	4.34	1.57-11.99	0.005	4.86	1.76-13.41	0,002
>75	791 (41.9)	10,11	3.65 - 28.0	<0.001	10.91	3.94 - 30.2	<0.001
Main outpatient clinic							
Coronary artery disease	1243 (21.7)	—	—		—	—	
Hypertension	611 (22.5)	1.05	0.94-1.17	0.396	1.08	0.96-1.20	0.208
Valvular heart disease	465 (23.3)	1.10	0.97-1.24	0.139	1.16	1.03-1.32	0,016
Dyslipidemias	382 (23.4)	1.10	0.97-1.26	0.144	1.12	0.98-1.28	0.089
Cardiomyopathies	516 (24.1)	1.15	1.02 - 1.29	0.023	1.27	1.13-1.43	<0.001
Arrhythmias	426 (24.5)	1.17	1.03-1.33	0.013	1.21	1.07-1.38	0.003
Other	954 (31.1)	1.63	1.48-1.80	<0.001	1.56	1.41 - 1.73	<0.001
Total	4597 (24.2)						

CKD, chronic kidney disease; OR, Odds ratio; CI, confidence interval.

Table S4. Studies evaluating the prevalence of chronic kidney disease in patients with cardiovascular disease

Country	Year	Population	Number of participants	Age (years)	Males (%)	Diabetes mellitus (%)	Equation for eGFR estimation	Use of two creatinine dosages three months apart for CKD confirmation	eGFR <60 ml/min/1.73m^2	Proteinuria (%)	CKD (%)
Brazil[a]	2022	Cardiovascular diseases	36,651	72	51	-	CKD-EPI[13]	No	31	28	39
		Coronary artery disease	11,599	72	66	-	CKD-EPI[13]	No	30	29	39
		Valvular heart disease	5,202	70	42	-	CKD-EPI[13]	No	27	32	38
		Cardiomyopathies	3,462	69	46	-	CKD-EPI[13]	No	32	23	37
		Arrhythmias	3,711	72	47	-	CKD-EPI[13]	No	31	24	40
Poland[1]	2020	Valvular heart disease	1,205	67	56	22	CKD-EPI[13]	Yes	37	-	-
Ethiopia[2]	2019	Diseases Cardiovascular	163	42	44	-	CKD-EPI[13]	No	24	25	-
Thailand[3]	2017	Hypertension artery	28,770	63	37	52	CKD-EPI[13]	No	38	62	-
Europe multinational[4]	2017	Coronary artery disease	7,998	65	76	27	CKD-EPI[13]	No	21	20	38
Global multinational[5]	2016	High risk	4,284	50	38	17	CKD-EPI[13]	No	8	15	36
Spain[6]	2010	Cardiovascular diseases	2,608	68	46	60	MDRD[14]	No	28	-	-

Country	Year	Population	Number of participants	Age (years)	Males (%)	Diabetes mellitus (%)	Equation for eGFR estimation	Use of two creatinine dosages three months apart for CKD confirmation	eGFR <60 ml/min/1.73m^2	Proteinuria (%)	CKD (%)
United States[7]	2009	Coronary artery disease	3,803	63	45	23	MDRD[14]	No	21	-	-
China[8]	2007	Coronary artery disease	3,513	69	66	33	MDRD[14]	No	25	8	-
Ireland[9]	2007	Cardiovascular diseases	1,272	66	72	12	MDRD[14]	No	36	-	-
Spain[10]	2006	Hypertension and obesity	4,585	62	48	33	MDRD[14]	No	23	-	-
United States[11]	2005	Hypertension	5,897	60	50	11	MDRD[14]	No	-	15	19
United States[12]	2003	Coronary artery disease	4,584	63	67	29	Cockcroft-Gault[15]	No	24	-	-

[a]Results of this study. eGFR, estimated glomerular filtration rate; CKD, chronic kidney disease, defined by eGFR < 60ml/min/1.73m^2 and/or proteinuria; Proteinuria, proteins in urinalysis 1+ or higher or albumin/creatinine ratio in spot urine sample >30mg/g; CKD-EPI, Chronic Kidney Disease Epidemiology Collaboration; MDRD, Modification of Diet in Renal Disease

References

1. Kuźma, Ł.; Małyszko, J.; Bachórzewska-Gajewska, H.; Niwińska, M.M.; Kurasz, A.; Zalewska-Adamiec, M.; Kożuch, M.; Dobrzycki, S. Impact of chronic kidney disease on long-term outcome of patients with valvular heart defects. *Int. Urol. Nephrol.* **2020**, *52*, 2161–2170.
2. Chala, G.; Sisay, T.; Teshome, Y. Chronic Kidney Disease And Associated Risk Factors Among Cardiovascular Patients. *Int. J. Nephrol. Renov. Dis.* **2019**, *12*, 205–211.
3. Krittayaphong, R.; Rangsin, R.; Thinkhamrop, B.; Hurst, C.; Rattanamongkolgul, S.; Sripaiboonkij, N.; Wangworatrakul, W. Prevalence of chronic kidney disease associated with cardiac and vascular complications in hypertensive patients: A multicenter, nation-wide study in Thailand. *BMC Nephrol.* **2017**, *18*, 115. https://doi.org/10.1186/si 2882-017- 0528-3.
4. Wagner, M.; Wanner, C.; Kotseva, K.; Wood, D.; De Bacquer, D.; Rydén, L.; Störk, S.; Heuschmann, P.U.; EUROASPIRE IV Investigators. Prevalence of chronic kidney disease and its determinants in coronary heart disease patients in 24 European countries: Insights from the EUROASPIRE IV survey of the European Society of Cardiology. *Eur. J. Prev. Cardiol.* **2017**, *24*, 1168–1180. https://doi.org/10.1177/2047487317708891.
5. Ene-Iordache, B.; Perico, N.; Bikbov, B.; Carminati, S.; Remuzzi, A.; Perna, A.; Islam, N.; Bravo, R.F.; Aleckovic-Halilovic, M.; Zou, H.; et al. Chronic kidney disease and cardiovascular risk in six regions of the world (ISN-KDDC): A cross-sectional study. *Lancet Glob. Health* **2016**, *4*, e307–e319. https://doi.org/10.1016/s2214-109x(16)00071-1.
6. Cases Amenós, A.; González-Juanatey, J.R.; Conthe Gutiérrez, P.; Matali Gilarranz, A.; Garrido Costa, C. Prevalence of chronic kidney disease in patients with or at a high risk of cardiovascular disease. *Rev. Esp. Cardiol*. **2010**, *63*, 225–228. https://doi.org/. https://doi.org/10.1016/sl 885-5857(10)70041-4.
7. McClellan, W.M.; Newsome, B.B.; McClure, L.A.; Cushman, M.; Howard, G.; Audhya, P.; Abramson, J.L.; Warnock, D.G. Chronic Kidney Disease Is Often Unrecognized among Patients with Coronary Heart Disease: The REGARDS Cohort Study. *Am. J. Nephrol.* **2009**, *29*, 10–17. https://doi.org/10.1159/000148645.
8. Liu, H.; Yu, J.; Chen, F.; Li, J.; Hu, D. Inpatients with coronary heart disease have a high prevalence of chronic kidney disease based on estimated glomerular filtration rate (eGFR) in China. *Heart Vessel.* **2007**, *22*, 223–228. https://doi.org/10.1007/s00380-006-0964-7.
9. Glynn, L.G.; Reddan, D.; Newell, J.; Hinde, J.; Buckley, B.; Murphy, A. Chronic kidney disease and mortality and morbidity among patients with established cardiovascular disease: A West of Ireland community-based cohort study. *Nephrol. Dial. Transplant.* **2007**, *22*, 2586–2594. https://doi.org/10.1093/ndt/gfm222.
10. Gomez, P.; Ruilope, L.M.; Barrios, V.; Navarro, J.; Prieto, M.A.; Gonzalez, O.; Guerrero, L.; Zamorano, M.A.S.; Filozof, C.; FATH Study Group. Prevalence of Renal Insufficiency in Individuals with Hypertension and Obesity/Overweight: The FATH Study. *J. Am. Soc. Nephrol.* **2006**, *17* (Suppl. 3), S194–S200. https://doi.org/10.1681/asn.2006080914.
11. Kramer, H.; Luke, A.; Bidani, A.; Cao, G.; Cooper, R.; McGee, D. Obesity and Prevalent and Incident CKD: The Hypertension Detection and Follow-Up Program. *Am. J. Kidney Dis.* **2005**, *46*, 587–594. https://doi.org/10.1053/j.ajkd.2005.06.007.
12. Reddan, D.N.; Szczech, L.A.; Tuttle, R.H.; Shaw, L.K.; Jones, R.H.; Schwab, S.J.; Smith, M.S.; Califf, R.M.; Mark, D.B.; Owen, W.F. Chronic Kidney Disease, Mortality, and Treatment Strategies among Patients with Clinically Significant Coronary Artery Disease. *J. Am. Soc. Nephrol.* **2003**, *14*, 2373–2380. https://doi.org/10.1097/01.asn.0000083900.92829.f5.
13. Levey, A.S.; Stevens, L.A.; Schmid, C.H.; Zhang, Y.L.; Castro, A.F., III.; Feldman, H.I.; Kusek, J.W.; Eggers, P.; Van Lente, F.; Greene, T.; et al. A New Equation to Estimate Glomerular Filtration Rate. *Ann. Intern. Med.* **2009**, *150*, 604–612. https://doi.org/10.7326/0003-4819-150-9-200905050-00006.
14. Levey, A.S.; Bosch, J.P.; Lewis, J.B.; Greene, T.; Rogers, N.; Roth, D. A more accurate method to estimate glomerular filtration rate from serum creatinine: a new prediction equation. Modification of Diet in Renal Disease Study Group. *Ann. Intern. Med.* **1999**, *130*, 461–470. https://doi.org/10.7326/0003-4819-130-6-199903160-00002.
15. Cockcroft, D.W.; Gault, M.H. Prediction of creatinine clearance from serum creatinine. *Nephron* **1976**, *16*, 31–41.
